# BioLQM: A Java Toolkit for the Manipulation and Conversion of Logical Qualitative Models of Biological Networks

**DOI:** 10.3389/fphys.2018.01605

**Published:** 2018-11-19

**Authors:** Aurélien Naldi

**Affiliations:** Computational Systems Biology Team, Institut de Biologie de l'École Normale Supérieure, École Normale Supérieure, CNRS, INSERM, PSL Université, Paris, France

**Keywords:** qualitative modeling, computational systems biology, biological networks, boolean networks, static analysis, model conversion

## Abstract

Here we introduce bioLQM, a new Java software toolkit for the conversion, modification, and analysis of Logical Qualitative Models of biological regulatory networks. BioLQM provides core modeling operations as building blocks for the development of integrated modeling software, or for the assembly of heterogeneous analysis workflows involving several complementary tools. Based on the definition of multi-valued logical models, bioLQM implements import and export facilities, notably for the recent SBML qual exchange format, as well as for formats used by several popular tools, facilitating the design of workflows combining these tools. Model modifications enable the definition of various perturbations, as well as model reduction, easing the analysis of large models. Another modification enables the study of multi-valued models with tools limited to the Boolean case. Finally, bioLQM provides a framework for the development of novel analysis tools. The current version implements various updating modes for model simulation (notably synchronous, asynchronous, and random asynchronous), as well as some static analysis features for the identification of attractors. The bioLQM software can be integrated into analysis workflows through command line and scripting interfaces. As a Java library, it further provides core data structures to the GINsim and EpiLog interactive tools, which supply graphical interfaces and additional analysis methods for cellular and multi-cellular qualitative models.

## 1. Introduction

Logical models are highly abstract dynamical models, which have been proposed to study biological regulatory systems in the late 60s (Kauffman, 1969; Thomas, 1973). This modeling framework has since gained popularity (Bornholdt, 2005; Saadatpour and Albert, 2013; Samaga and Klamt, 2013) and has been successfully applied to a wide range of regulatory and signaling systems (Saez-Rodriguez et al., 2007; Naldi et al., 2010; Helikar et al., 2013; Abou-Jaoudé et al., 2016).

In logical models, components are represented by discrete variables with a small range of possible values, representing qualitative differences in activity. Boolean components can only be active (1) or inactive (0), while multi-valued components define multiple activity levels. Regulatory effects are often represented as signed arcs between components in the **regulatory graph**. These effects are further formalized as logical rules (also called logical parameters or logical functions), specifying the target activity level of each component according to the current levels of its regulators (a subset of all model components). Interactive software for model definition such as GINsim (Naldi et al., 2018a) or The Cell Collective (Helikar et al., 2012) enable the definition of regulatory graphs and logical rules. However, these logical rules are self-contained and can be used to recover signed regulatory interactions. The relative simplicity of this formalism enables the definition of large models with dozens of components, without requiring precise knowledge of kinetic parameters. A formal definition of logical qualitative models is provided in Appendix 1 in Supplementary Material.

**Figure 1:**
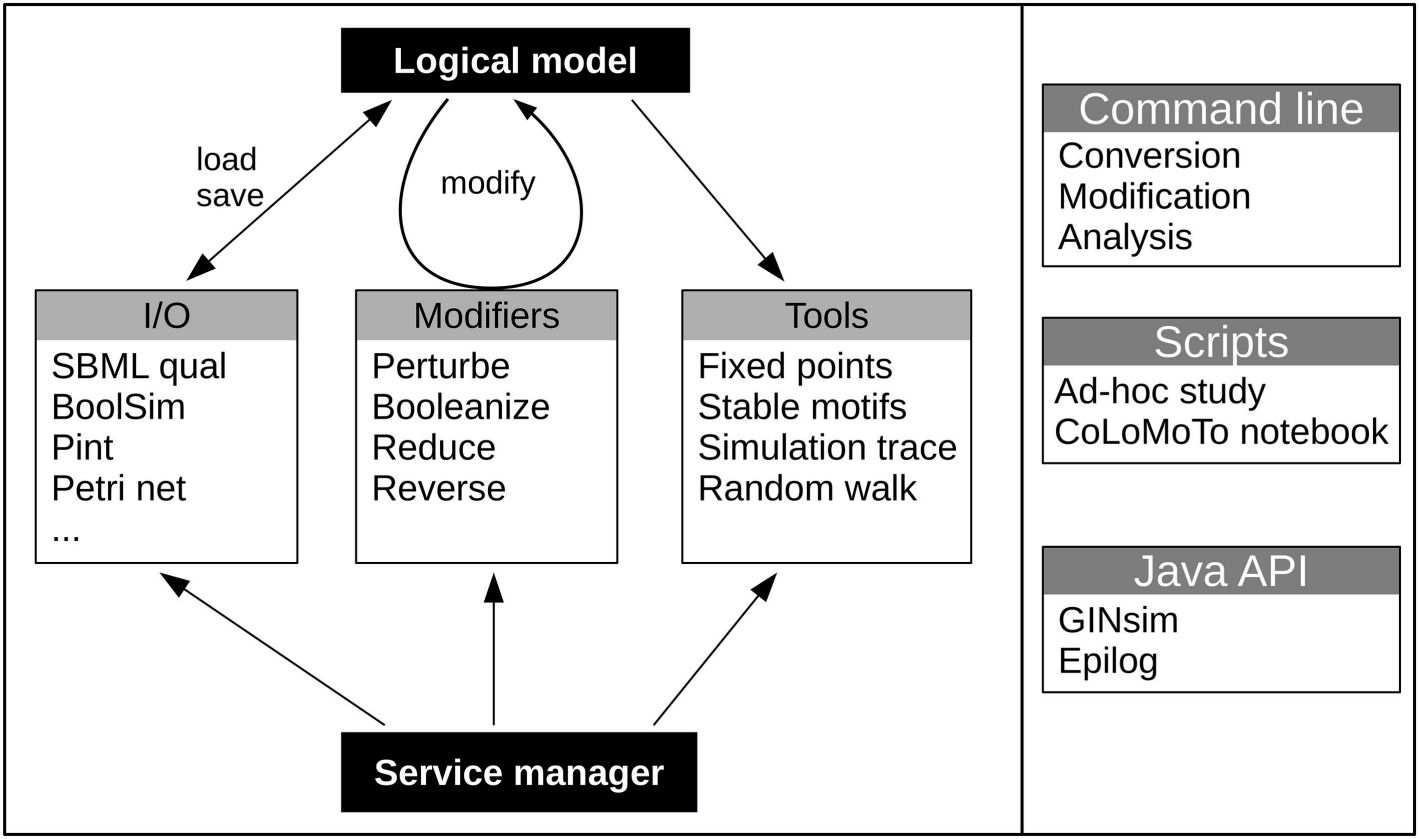
Global structure of the bioLQM toolkit. The bioLQM toolkit is centered around a data structure for the representation of logical qualitative models. Based on this data structure, (i) the I/O module contains a collection of formats enabling model loading and saving ; (ii) the modifiers module contains a collection of model modifiers to transform an input model into a modified model ; (iii) the tools module contains a collection of analysis tools. All these feature are accessible through a central service manager, which handles service discovery and serves as main entry point for the Java API. A simple command line launcher provides quick execution of simple workflows, while a scripting engine can be used for more complex use cases.

The CoLoMoTo consortium was recently founded to facilitate model sharing and foster cooperation in the qualitative modeling community, building on the introduction of the SBML qual exchange format (Chaouiya et al., 2013; Naldi et al., 2015). The bioLQM toolkit presented here reinforces this effort by implementing a collection of model modification, format conversion, and dynamical analysis operations in an extensible architecture illustrated in Figure 1. On one hand, format conversions enable the integration of several software tools in complex analysis workflows. On the other hand, the core data structure and model modifications provide building blocks for the development of integrated modeling tools, which can add their own model edition and visualization capabilities. BioLQM is notably embed in the popular GINsim software (Naldi et al., 2018a), which provides a graphical interface to most of its features. It is also used as backend for model definition and computation of successor states in Epilog (Varela et al., 2013), as well as in the CoLoMoTo notebook for model conversion and some dynamical analysis features (Naldi et al., 2018b). Preliminary versions of this toolkit were mentioned as the “LogicalModel” library Chaouiya et al. (2013) and Naldi et al. (2015).

Section 2 introduces model loading, saving and converting operations. Section 3 introduces the simulation and dynamical analysis features. Section 4 introduces model modifications. Section 5 illustrates the use of these features through the command-line and scripting interfaces for the analysis of a small model of the p53-Mdm2 network controlling DNA repair.

## 2. Loading and converting logical qualitative models

The increasing use of qualitative models to study biological systems led to the development of various software tools for the logical formalism (Albert et al., 2008; Garg et al., 2008; Müssel et al., 2010; Terfve et al., 2012; Naldi et al., 2018a) and related qualitative approaches (Batt et al., 2012; Paulevé, 2017; Stoll et al., 2017). Most software tools use their own file format for the definition of models, hindering the delineation of analysis workflows combining different tools. The SBML qual exchange format (Chaouiya et al., 2013) has recently been proposed to improve interoperability between modeling tools. However SBML support is often missing from existing software and may not be a priority for newer ones.

To ease model exchange between software tools that do not all support the SBML qual format, the bioLQM toolkit provides an extensible list of format handlers connected to the internal model representation. Each format is described as a Java class providing annotations (name of the format, default file extension and multi-valued support) along with optional implementations of model import (loading a file into the internal representation) or export (saving the internal representation to a file) operations. These descriptor classes are available through service discovery to facilitate the addition of new formats.

The supported formats are listed in Table 1 and in bioLQM documentation ^1^. BioLQM uses JSBML (Rodriguez et al., 2015) to load and save SBML qual models. The other import parsers are based on the antlr parser generator (Parr and Quong, 1995). While some formats natively support multi-valued models, many are limited to the Boolean case. Multi-valued models can be exported to these Boolean formats through an implicit booleanization step, described in section 4.

**Table 1:** Available formats.

**File extension**	**Multi-valued**	**Import**	**Export**	**Description and associated tools**
sbml	x	x	x	SBML qual Exchange format (Chaouiya et al., 2013)
bnet		x	x	(Py)BoolNet (Müssel et al., 2010; Klarner et al., 2017)
booleannet		x	x	booleannet (Albert et al., 2008)
boolfunction		x	x	Boolean functions
boolsim		x	x	boolsim (genYsis) (Garg et al., 2008)
cnet		x	x	BNS (Dubrova and Teslenko, 2011)
ginml	x		x	GINsim (Naldi et al., 2018a)
mnet	x	x	x	Custom text format for multi-valued models
tt	x	x	x	Truth table
an	x		x	Pint automata network (Paulevé, 2017)
apnn, pnml, ina	x		x	Conversion to Petri Net formats (Chaouiya et al., 2011)
gna	x		x	GNA (Piecewise-linear formalism) (Batt et al., 2012)
bnd			x	MaBoSS (Stochastic Boolean model) (Stoll et al., 2017)

*The Import/Export capabilities are listed in the corresponding columns (all formats can be exported). The formats natively supporting multi-valued models are also identified, other formats rely on implicit model booleanization*.

## 3. Model dynamics and simulation

A **state** of a model is a vector giving the activity levels of all its components. As the activity level of each component is restricted to a finite range, the **state space** (containing all possible states) itself is also finite. However, the total number of possible states grows exponentially with the number of components. We say that a component is **called to update** in a given state if the evaluation of the associated logical rule is different from its current activity level: for example an inactive component can become active. **Stable states** (also called fixed points, or steady states) are states in which no component is called to update. Such stable states denote a qualitative equilibrium in which all components can maintain their current activity level.

**Figure 2:**
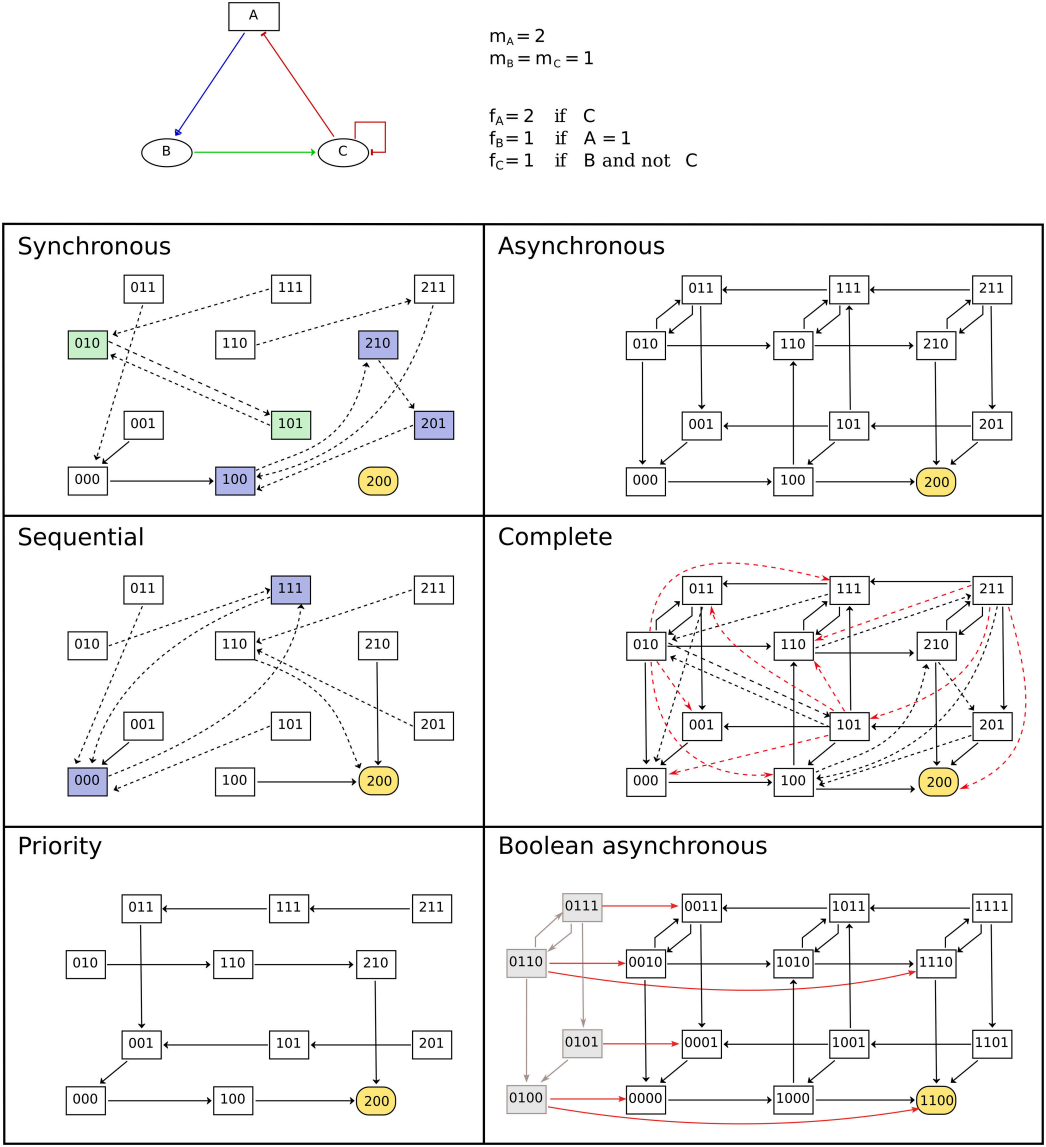
Comparison of updating modes. State transition graphs obtained with the multi-valued model shown in the top part using various deterministic (left-side) and non-deterministic (right-side) updatings. Dashed arcs denote multiple transitions and node coloring emphasizes attractors. Note that the stable state is common to all updating modes. The sequential and priority updaters follow the implicit ordering of the components. The STG obtained with the complete updating contains all synchronous and asynchronous transitions, as well as additional transitions to leave the states encompassing more than two updating calls. These transitions are colored in red in the corresponding panel. Finally, the bottom-right panel contains the asynchronous STG obtained for the booleanized version of the model. In this STG, gray nodes and arcs on the left side correspond to non-admissible states and transitions between them. These states are unreachable from the admissible ones, and the transitions enabling to leave this set of states are highlighted.

The dynamics of the model (i.e., its evolution over time) is given by transitions between states of the model, controlled by the updating calls (i.e., by the logical rules of the model) and by **updating modes** which define the synchronization between concurrent updating calls. Various types of updating modes have been introduced, with most software tools focusing on a specific subset. BioLQM aims to provide an extensive choice of updating modes in a single toolkit. In the following subsections, we further distinguish deterministic and non-deterministic simulations and provide an overview of all updating modes implemented in bioLQM. While stable states, which have no transition toward other states, do not depend on the updating mode, reachability properties and cyclical attractors can be strongly affected by the choice of updating mode as illustrated in Figure 2. More formal definitions of the updating calls and updating modes are given in Appendix 2 in Supplementary Material.

### 3.1. Deterministic simulations

In a **deterministic** simulation, each state has a unique successor, except stable states which have no successor at all as we consider here that a successor must denote a change of state. Starting with an initial state, a deterministic simulation yields an ordered list of successive states, called a **trace**. Given a sufficient number of steps, all traces end in an **attractor**, which can be either a stable state or a **cyclical attractor** of length *k* in which the *k*-th successor of each state is itself. The trace tool, illustrated in section 5, uses an initial state and a deterministic updater to compute a simulation trace. The following deterministic updating modes are supported:

- The **synchronous** (or parallel) updating applies all logical rules at the same time (Kauffman, 1969).
- The **sequential** updating applies all rules in a pre-determined order. Instead of evaluating all rules on the original state before updating all components at once as in the synchronous case, they are evaluated on the state obtained after applying the previous rule. The selected order can then change dramatically the successor state: a different sequential updater can be defined for each possible ordering.
- The **block-sequential** updating generalizes the sequential one by considering groups of components updated synchronously (Robert, 1986). The definition of a block-sequential updater relies on an ordered partition of the model components.
- The **synchronous priority** updating is also based on a partition of components into blocks, but only the first block containing updated components will be considered. The set of possible updaters is a subset of the priority-based updaters introduced by Fauré et al. (2006).

### 3.2. Non-deterministic simulations

In a **non-deterministic** simulation, each state can have several successors. Starting with an initial state, a non-deterministic simulation can lead to a large number of alternative trajectories. This type of dynamics if often represented as a **State Transition Graph** (STG), where the nodes are states of the model, and arcs denote possible transitions between these states. Like in the deterministic case, all trajectories end in an attractor, but starting from an initial state, a non-deterministic simulation can lead to several alternative attractors. These attractors can be **stable states**, **cyclical attractors**, as well as sets of intertwined cycles called **complex attractors**. More formally, all attractors are terminal strongly connected components of the STG. State transition graphs can represent deterministic traces as well as more complex dynamical behaviors. Such a graph can cover several alternative initial states or even all possible states. The current version of bioLQM supports the definition of non-deterministic updaters, enabling the computation of the lists of successor states. However, it does not provide a complete engine for non-deterministic simulations, or a data structure for state transition graphs. GINsim (Naldi et al., 2018a) implements these features on top of bioLQM. The following non-deterministic updating modes are supported:

- The **asynchronous** updating applies all logical rules independently. All successors of a state change exactly one component (Thomas, 1973).
- The **complete** updating considers all possible combination of components to be updated at once. The set of successors includes all asynchronous successors, as well as the synchronous one (and more).
- The **priority** updating generalizes the *synchronous priority* introduced above by allowing some of the blocks (priority classes) to be updated asynchronously (Fauré et al., 2006).

### 3.3. Stochastic simulations

Stochastic updaters enable the computation of a single successor, which is selected randomly among multiple possibilities and can thus change between calls. A stochastic updater can be derived from any non-deterministic updater by assigning identical probabilities to all transitions defined by the original updater. Alternatively, a custom updater can be constructed by defining individual probabilities.

BioLQM provides the random tool to compute single random trajectories using the above stochastic updaters. This tool is limited to the construction of individual trajectories and does not provide a complete stochastic analysis. As listed in Table 1, bioLQM enables the conversion of Boolean models to the format of the MaBoSS software, which uses the Gillespie algorithm to estimate the probabilities of Boolean states of a continuous time Markov process, and provides a collection of scripts to further analyze the simulation results (Stoll et al., 2017).

### 3.4. Identification of attractors

The dynamical analysis of large regulatory networks through model simulation suffers from combinatorial explosion, especially in the non-deterministic case. BioLQM implements two published methods based on constraint-solving for the identification of attractors without explicit state enumeration.

1. The first method enables the identification of **stable states** (fixed points) by extracting and combining stability conditions from the logical rules (Naldi et al., 2007). BioLQM includes this implementation, using decision diagrams to manipulate stability conditions, and introduces an alternative implementation based on the clingo ASP solver (Gebser et al., 2011), which tends to be slower for small models, but can scale better in some cases. Similar methods are also available in the GNA and Pint tools (Batt et al., 2012; Paulevé, 2017).
2. The efficient identification of cyclical attractors and complex attractors remain a challenging problem, especially as these attractors can depend on the updating mode. **Stable patterns** have recently been proposed as an approximation of complex attractors, which can be identified efficiently and does not depend on the updating mode (Zañudo and Albert, 2013; Klarner et al., 2014). Here, a pattern is a partially-defined state where some components have a fixed activity level, while others are undefined. Such a pattern represents all states with matching activity levels for the defined components (i.e., 2^*k*^ possible states for *k* undefined Boolean components). A pattern is stable if the images of all included states belong to the pattern (the image of a state is its successor in a synchronous updating). BioLQM proposes an adapted version of the method implemented in PyBoolNet (Klarner et al., 2014, 2017) using the clingo ASP solver (Gebser et al., 2011), and introduces a new alternative implementation based on decision diagrams.

While complex attractors are well estimated through stable patterns, their exact identification requires further analysis using external software tools, adapted to the selected updating mode. In the synchronous case, the BNS tool (Dubrova and Teslenko, 2011) identifies cyclical attractors of length *k* using constraint solving. This approach could be extended to other deterministic updatings, but can not handle non-deterministic cases. In contrast, BoolSim uses symbolic exploration for the identification of complex attractors in the synchronous and asynchronous case (Garg et al., 2008). While this approach scales better than simple simulation, it is more sensitive to combinatorial explosion than approaches based on constraint-solving. To perform the analysis provided by the BoolSim and BNS tools, bioLQM can convert models to their respective formats.

## 4. Model modifications

Several software tools propose to emulate biological **mutations** by constructing model variants in which one or several logical rules have been modified. In bioLQM, the various **model modification** tools enable the flexible definition of model variants. The resulting modified models can have a different set of components than the original model. Each modification can be described by a keyword (identifier of the type of modification) and some parameters. The model modifier API in bioLQM allows to chain several modifications before model conversion or analysis. The following describes the various types of model modifications implemented in bioLQM.

### Perturbations

A **perturbation** (often called **mutation**) enables to change some of the logical rules of a model. BioLQM provides three types of “atomic perturbations” (fixed value, range restriction, and removal of a regulator) which modify a single logical rule. They are briefly described below, more formal definitions can be found in Appendix 3 in Supplementary Material. “Multiple perturbations” can then be used to combine several atomic perturbations. The definition of these perturbations is supported by a simple syntax, as illustrated in section 5.3 and described in the online documentation.

Perturbations are commonly used to model gene knockouts by **fixing the activity level** of the corresponding component to 0, or ectopic expressions by fixing it to 1. Multi-valued components can also be fixed to a higher activity level (inside their normal activity range).

**Restricting the activity range** of multi-valued components enables to account for a partially impaired activity ([loss of the higher activity level(s)] or to set a minimal activity level.

Lastly, it is possible to define the **perturbation of a single interaction**, i.e., to remove one of the regulators of a component. This type of perturbation enables for example the definition of the loss of a single binding site preventing the action of the source component on a subset of its targets. The removal of an interaction amounts to rewrite the logical rule of its target component. Note that the atomic perturbation describes the effect on a single target: a single “biological mutation” may correspond to a “multiple perturbation” in the model if several targets are affected by the loss of the same binding site. This type of perturbation is also convenient to evaluate the importance of an interaction representing an hypothetical effect.

### 4.1. Model reduction

Model reduction aims to ease the analysis of models with a large number of components by constructing a smaller model involving fewer components, but exhibiting similar dynamical properties. BioLQM provides a model reduction method which updates the logical rules of the remaining components to emulate the effect of the removed components (Naldi et al., 2011; Veliz-Cuba, 2011). This reduction preserves key dynamical properties of the model, in particular the stable states and stable patterns. However, it can affect some dynamical properties, depending on the choice of reduced components.

This modifier usually relies on the specification of the set of components to reduce. Some types of reduction can be fully automated. In particular, bioLQM supports the reduction of output components, which was shown to preserve attractors and reachability properties (Naldi et al., 2012), as well as the propagation of fixed components, which has also been shown to preserve attractors (Saadatpour et al., 2013).

After reduction, the reduced components are not fully eliminated from bioLQM: they are no longer allowed to regulate other components, but they keep a logical rule to allow the computation of their expected value in the reduced model.

### 4.2. Boolean mapping of multi-valued models

As discussed above, some software tools and formats are limited to Boolean models, for example as they rely on specific theoretical results or data structures. To apply such software tools to the analysis of a multi-valued model, we can construct a Boolean model such that its dynamical properties can be transferred to the original multi-valued model.

This **model Booleanization** step is based on the Boolean mapping discussed by Didier et al. (2011). In this mapping, a multi-valued component with a maximal activity level *m* is replaced by *m* Boolean components, each denoting increasing activity. All possible states of the original model can then be associated to states of the Boolean model. The logical rules of the new model ensure that we obtain the same transitions between these states. However, some states of the Boolean model are not mapped to states of the original model. These additional states are called “non-admissible states.”

The dynamical properties observed on the admissible states of the Boolean model can be transferred to the original model. The implementation proposed here further ensures that all synchronous and asynchronous simulations starting with a non-admissible state can lead to an admissible state after a sufficient number of steps. This property ensures that no attractor contains any non-admissible state (see Figure 2).

Model Booleanization is used automatically when converting multi-valued models to formats supporting only Boolean models. It can also be performed explicitly, like other model modifications.

## 5. Use case: analysis of the p53-Mdm2 network

The cellular response to DNA damage relies on the p53 transcription factor, which induces the synthesis of DNA repair proteins. The ubiquitin ligase Mdm2 blocks the transcriptional activity on p53 in the nucleus, while p53 activates the transcription of Mdm2 and inhibits its nuclear translocation. In this section, we use a logical model involving DNA damage, p53, Mdm2 in the cytoplasm and Mdm2 in the nucleus. See the recently published GINsim tutorial Naldi et al. (2018a) and the enclosed references for a more complete description of this system and its encoding into a logical model.

In the following, we define the model in a text file named p53.mnet, using a simple text format for the definition of multi-valued logical models (p53 and cytoplasmic Mdm2 are represented by ternary components). Each line of the file reproduced below assigns a logical function to one of the components of the model. The line starts with the the identifier of the component, separated from the function itself by a leftwards arrow ( < -). The &, |, and ! symbols stand for the AND, OR, and NOT operations respectively. The colon character (:) is used to specify multi-valued thresholds, both for assigning the target component and inside the functions.


DNAdam      <-   DNAdam   &   !p53:2
p53:2       <-   !Mdm2nuc
Mdm2cyt:1   <-   !p53:2
Mdm2cyt:2   <-   p53:2
Mdm2nuc     <-   Mdm2cyt:2   |   (Mdm2cyt   &   !p53   &   !DNAdam)

### 5.1. Install and launch bioLQM

Documentation, source code and releases (under the LGPL v3 license) are available on http://colomoto.org/biolqm. BioLQM is distributed as a JAR file^2^, which can be launched with the command java -jar bioLQM.jar. In this section, we will use the bioLQM command as shorthand.

### 5.2. Resting state and DNA repair

We start by looking for the stable states (fixed points) of this model. For this, we launch bioLQM on the command-line, load the model from the p53.mnet file defined above, and run the fixpoints tool. The corresponding command line and its output are reproduced hereafter.

**$  bioLQM  p53.mnet  -r  fixpoints**
DNAdam  p53  Mdm2cyt  Mdm2nuc
0011

In the output, bioLQM displays a first line with the list of components, followed by a line for each identified stable state, giving the activity level of each component in the same order. The p53-Mdm2 model has a single stable state corresponding to a resting state in absence of DNA damage. In this state, the basal activity of Mdm2 prevents p53 activation. This analysis shows all the stable states of the model, but does not identify more complex attractors. We can then use the trapspace tool to identify stable patterns, which provide a good approximation of complex attractors in practice.

**$  bioLQM  p53.mnet  -r  trapspace**
DNAdam  p53_b1  p53_b2  Mdm2cyt_b1  Mdm2cyt_b2  Mdm2nuc
0  0  0  1  0  1

In this output, the two multi-valued components of the model have been extended to four Boolean components. While this requires a careful interpretation, it provides fine-grained results for complex attractors in which multi-valued components can be restricted to a range of their possible activity levels. Here we obtain a single pattern corresponding to the previously identified stable state. Note that this result does not strictly rule out the existence of a complex attractor, but attractors which do not correspond to such stable patterns are rare in practice and often depend on subtle delay effects. In this model, the resting state is indeed the only attractor.

We can then evaluate the behavior of this network upon addition of DNA damage to this resting state. For this, we use the trace tool to perform a synchronous simulation, starting with an initial state (defined after the -i flag) obtained by adding DNA damage to the resting state.

**$  bioLQM  p53.mnet  -r  trace  -i  1011**
1011
1010
1110
1210
0220
0221
0121
0011

In this simulation trace, we see that the introduction of DNA damage in the resting state leads to the inactivation of Mdm2 in the nucleus, enabling the activation of p53. This triggers DNA repair and allows Mdm2 to accumulate in the cytoplasm. Finally, Mdm2 can enter the nucleus and inhibit p53, coming back to the resting state. In this simulation, we assume that all possible transitions happen synchronously in each state, which could lead to artefactual trajectories. Asynchronous simulations are widely considered as more reliable, but they lead to a large number of alternative branches and are not well suited for simple command-line simulations. We can however perform a random walk in the set of possible asynchronous trajectories using the random tool. In this case, all asynchronous trajectories eventually lead to the same stable state (not illustrated here).

### 5.3. Definition of model perturbation

We then apply a perturbation to study the impact of a p53 knockout on the list of stable states. The -m perturbation parameters trigger the construction of a modified model. The following parameters (up to the next flag starting with a minus sign) define the modified functions. Here p53%0 describes a loss of p53 activity.

**$  bioLQM  p53.mnet  -m  perturbation  p53%0  -r  fixpoints**
DNAdam  p53  Mdm2cyt  Mdm2nuc
0011
1010

We see that the resting state is still valid in the p53 knockout, however a new stable state appears in which DNA damage could not be repaired.

Instead of a full knockout of p53, we then evaluate a more subtle perturbation in which only its ability to trigger the DNA repair machinery is impaired. This corresponds to the removal of the interaction between p53 and DNAdam in our model.

**$  bioLQM  p53.mnet  -m  perturbation  p53:DNAdam%0  -r  fixpoints**
DNAdam  p53  Mdm2cyt  Mdm2nuc
0011

**$  bioLQM  p53.mnet  -m  perturbation  p53:DNAdam%0  -r  trapspace**
DNAdam  p53_b1  p53_b2  Mdm2cyt_b1  Mdm2cyt_b2  Mdm2nuc
0  0  0  1  0  1
1  -  -  1  -  -

Here we see that this perturbation does not affect the stability of the resting state, and does not create an additional stable state as in the full p53 knockout case. However, the trapspace tool reveals the creation of a complex attractor involving oscillations of p53 and Mdm2. Note that these oscillations exist transiently in the original model but lead back to the resting state after DNA repair.

### 5.4. Model conversion enables interoperability

As discussed in section 2, the analysis of complex models can combine several software tools. After running the following command, the new p53.sbml file will contain the functions defined above in the SBML qual format. This format is suitable for use in several other tools, or for submission in the BioModels database (Chelliah et al., 2013).

**$  bioLQM  p53.mnet  p53.sbml**

### 5.5. Definition of complex analysis as scripts

More complex analysis tasks can use the integrated **scripting interface**. Based on the java scripting engine, it supports scripts written in javascript (as part of the java platform) or in another supported language by providing additional libraries (including python and lua). The following sample script generates all possible individual knockout perturbations, and saves each modified model.


filename  =  lqm.args[0]
model  =  lqm.load(filename)
nodes  =  model.getComponents()
for (i in nodes)  {
    node = nodes[i]
    perturbed = lqm.modify(model, ′perturbation′, node+′%0′)
    lqm.save(perturbed, filename+"_"+node+"_KO.mnet")
}

This script can be launched using the -s flag, followed by the script file name. Additional arguments can be used to adapt the behavior of the script. In this example, we specify the name of the original model file.


$ bioLQM -s generate_perturbations.js p53.mnet

The recently introduced CoLoMoTo Docker image (Naldi et al., 2018b) provides a python API integrating several complementary software tools. This environment includes a dedicated python API for bioLQM, which plays a central role in model conversion.

## 6. Summary and discussion

The increasing use of logical models of biological regulatory networks led to the development of multiple complementary software tools for their analysis. The recent introduction of the SBML qual format (Chaouiya et al., 2013) and the formation of the CoLoMoTo consortium (Naldi et al., 2015) aims to facilitate the exchange of models between tools. The bioLQM toolkit enables the use of additional software tools through conversion to their native formats. It provides model conversion operations in the CoLoMoTo notebook (Naldi et al., 2018b), enabling the delineation of analysis workflow involving a series of different tools.

BioLQM can also be used to apply various perturbations to the converted models, enabling the study of model variants emulating a knockout, an ectopic activity, or the loss of an interaction. Model modifications include the booleanization of multi-valued models for analysis with tools restricted to a Boolean formalism, as well as model reduction, decreasing the number of components to ease the analysis of complex models.

Finally, bioLQM provides several internal tools for the dynamical analysis of logical models. Two of the included tools allow the construction of deterministic and stochastic simulation traces, based on a comprehensive collection of updating modes. BioLQM also implements non-deterministic updating modes, which can be used as core components of complete simulation engines, as done by the GINsim (Naldi et al., 2018a) and Epilog (Varela et al., 2013) software suites. Two other tools enable the efficient identification of stable states and the approximation of most complex attractors.

The features described above are organized in a flexible architecture to facilitate the addition of new modules (file formats, model modifications, analysis tools) and to provide a consistent API. In the next version, the configuration API of analysis tools will be further improved to improve their use through python scripts in the new CoLoMoTo notebook.

Hardware requirements strongly depend on the size and structure of models and the operations performed. The complexity of individual logical rules can be a limiting factor: components with tens of regulators could have intricate rules with high computational cost. Fortunately, such rules are seldom used in biological models. Any desktop computer should be able to load and convert most models, including large ones. However, detailed dynamical analysis of models beyond 30 components can rapidly fill the available memory. In bioLQM, the fixpoints and trapspace analysis tools rely on efficient constraint-solving methods, which can scale to hundreds of components. The trace and random simulation tools are designed to work on large models as well by avoiding to store all visited states and interrupting the simulation when a stable state is reached or after a limit on the number of steps. In future versions, these tools will further use the identified trapspaces to interrupt the simulation when reaching a complex attractor.

BioLQM uses decision diagrams to store the logical rules internally, which enforces a normalized representation of the function, depending on the ordering of components. It has the advantage of providing guarantees on the number of tests to perform to evaluate a function, but it replaces the original representation of the function, making it harder to manipulate by the user afterwards. Future version will include several alternative representations to preserve hand-crafted logical functions through conversion (when the output format allows it).

Logical models are non-deterministic when using the asynchronous updating, however individual logical rules are deterministic: they associate a single “target value” to each state of the system. The ability to lift this limitation is considered in the design of the new internal data structure, but is not an immediate goal: the next releases of bioLQM will remain focused on deterministic functions.

In logical models (and by extension in bioLQM), each component is associated to its own logical rule, however the Petri net and automata network formalisms separate components from transitions. This separation allows in particular the definition of transitions affecting several components simultaneously. Such behaviors could be emulated in logical models through the addition of synchronizing components. Proper support for this use case would require extensions of the SBML qual specification, as well as changes in the internal data structure.

Like most modeling tools, bioLQM is currently centered on logical rules, however a complete model may contain important additional information, such as annotations and graphical layout information. Model annotations are supported in SBML core (without additional extensions), however annotations can be defined in any format, hindering interoperability. Further discussions are needed within the community to delineate best practices and ensure that annotations can be shared efficiently. Graphical layout information can be stored along with SBML qual models using a dedicated extension. This information is currently supported by JSBML and GINsim, it will be integrated in future versions of bioLQM. JSON “sidecar” files could then be used to facilitate the integration of such additional information with file formats which do not support it directly.

The reproducibility of model analysis relies on sharing both the model itself and the definition of simulation parameters, in particular initial states and updating modes. A single initial state can be defined in the SBML qual file. Additional initial states and simulation parameters fall in the scope of the Simulation Experiment Description Markup Language (SED-ML) format (Bergmann et al., 2015), which does not yet support qualitative models. Ongoing discussions should lead to extensions of the SED-ML format and the Kinetic Simulation Algorithm Ontology (Courtot et al., 2014) to describe model modifications and simulation parameters. These extensions will then be integrated into bioLQM and other qualitative modeling software.

## Author contributions

The author confirms being the sole contributor of this work and has approved it for publication.

### Conflict of interest statement

The author declares that the research was conducted in the absence of any commercial or financial relationships that could be construed as a potential conflict of interest.

## Footnotes

^1^See http://colomoto.org/biolqm/

^2^It requires a Java Runtime Environment, see https://www.java.com
