# Supplementary material for: BioLQM: A Java Toolkit for the Manipulation and Conversion of Logical Qualitative Models of Biological Networks
Source: Front Physiol. 2018 Nov 19;9:1605. doi: 10.3389/fphys.2018.01605 (PMC6254088; doi:10.3389/fphys.2018.01605)
Supplement: Supplementary file 1 [file Presentation_1.pdf]

[Appendix] BioLQM: a java toolkit for the manipulation and conversion of Logical Qualitative Models of biological networks

Aurélien Naldi[1,*]

[1] *Computational Systems Biology Team, Institut de biologie de l'École normale supérieure (IBENS),*
École normale supérieure, CNRS, INSERM, PSL Université Paris, France,

Correspondence*:
Aurélien Naldi
aurelien.naldi@ens.fr

This document contains some formal definitions of the methods implemented in the bioLQM software. It completes the technology report published in *frontiers in Physiology*, which provides a general introduction to the formalism and software.
The main document is available with DOI: 10.3389/fphys.2018.01605.
The bioLQM software is available at `http://www.colomoto.org/biolqm`.

1 LOGICAL QUALITATIVE MODEL

A **Logical Qualitative Model** $\mathcal{L} = (\mathcal{G}, \mathcal{M}, \mathcal{F})$ with n components is defined by:

- $\mathcal{G} = \{g_i\}_{i=1...n}$, the set of its components.
- $\mathcal{M} = \{m_i \in \mathbb{N}^*\}_{i=1...n}$, the maximal activity levels of these components,
  $\mathcal{S}_i = [0, m_i]$ is the activity range of the component g_i, and $\mathcal{S} = \prod \mathcal{S}_i$ is the state space.
- $\mathcal{F} = \{f_i : \mathcal{S} \to \mathcal{S}_i\}_{i=1...n}$, the logical functions defining its dynamical behavior.

A **Boolean model** $(\mathcal{G}, \mathcal{F})$ is a logical qualitative model such that all $m_i = 1\ \forall i \in [1, n]$ (*i.e.* $\mathcal{S} = [0, 1]^n$).

2 STATE SPACE AND DYNAMICS

In the following, we define the state and dynamics of a model, which can be computed from its logical functions f_i. For simplicity, we use i as a shorthand for g_i when no ambiguity is possible.

A (qualitative) state $x \in \mathcal{S}$ of the model is a vector giving the activity levels of all components, where x_i denotes the activity of the component i: $x = (x_i \in \mathcal{S}_i)_{i=1...n}$. A component i is **called to update** in the state x if $f_i(x) \neq x_i$. The state x is a **stable state** (also called fixed point, or steady state) if no component is called to update, *i.e.* if $x_i = f_i(x)\ \forall i \in 1 \ldots n$.

We define unitary update functions $u_i : \mathcal{S} \to \mathcal{S}_i$ such that $u_i(x) = x_i + \Delta_i(x)$, where

$$\Delta_i(x) = \begin{cases} 0 & \text{if } f_i(x) = x_i \\ 1 & \text{if } f_i(x) > x_i \\ -1 & \text{if } f_i(x) < x_i \end{cases}.$$

In the Boolean case, these unitary functions are identical to the logical functions f_i by construction. They enforce unitary transitions in the multi-valued case.

We call $f(x) = (f_i(x))_{i=1...n}$ the **image** of the state x, and $u(x) = (u_i(x))_{i=1...n}$ its **unitary image**.

Given a state $x \in \mathcal{S}$ and a subset of the components $p \subset \mathcal{G}$, let $\overline{x}^p$ be the state where all the components of the subset have been updated at once:

$$\overline{x}_i^p = \begin{cases} u_i(x) & \text{if } i \in p \\ x_i & \text{otherwise} \end{cases}.$$

For simplicity, $\overline{x}^i$ denotes $\overline{x}^{\{i\}}$.

Deterministic updatings

- In the **synchronous** updating, the unique successor of x is its unitary image $u(x)$.
- In the **sequential** updating following the default order of components, the unique successor of the state x is $(u_n \circ \cdots \circ u_1)(x)$. A different sequential updater can be defined for each possible ordering of $\mathcal{G}$.
- A **block-sequential** updater is based on an ordered partition of $\mathcal{G}$ in m non-overlapping subsets $(m \leq n)$. Let $\mathcal{P} = (p_1, \ldots, p_m \mid p_k \subset \mathcal{G}\ \forall k)$ be this ordered partition. The function $v_k : \mathcal{S} \to \mathcal{S}$ such that $v_k(x) = \overline{x}^{p_k}$ updates all components of the subset p_k synchronously. The unique successor of the state x by the block-sequential updater defined by the ordered partition $\mathcal{P}$ is then given by combining these functions: $(v_m \circ \cdots \circ v_1)(x)$.
- The **synchronous priority** updating is based on the partitioning $\mathcal{P}$ defined for the block-sequential case, but only the first updated subset is considered. If the state x is not a stable state, then there exists k such that the subset p_k is the first one containing updated components: $v_k(x) \neq x$ and $v_i(x) = x\ \forall i < k$. Then $v_k(x)$ is the successor of x in this updating. Note that this type of updating is the deterministic subset of the non-deterministic priority updatings defined below.

Non-deterministic updatings

- In the **asynchronous** updating, all logical functions are applied independently and all successors of the state x differ from x by exactly one component: the set of successors of x is $\{\overline{x}^i \neq x\ \forall i \in \mathcal{G}\}$.
- In the **complete** updating, any number of components can be updated at once: the set of successors of x is $\{\overline{x}^p \neq x\ \forall p \subset \mathcal{G}\}$. This set of successors includes all asynchronous successors, as well as the synchronous one.
- The **priority** updater generalizes the *synchronous priority* defined above by considering asynchronous updates between the blocks (priority classes). Starting with $\mathcal{P} = (p_1, \ldots, p_m \mid p_k \subset \mathcal{G}\ \forall k)$, the ordered partition of $\mathcal{G}$ defined above, each subset p_k is further partitioned in a_k subsets $p_{k,1}, \ldots, p_{k,a_k}$. If the state x is not a stable state, then there exists k such that the subset p_k is the first one containing updated components: $v_k(x) \neq x$ and $v_i(x) = x\ \forall i < k$. Then $v_{k,1}(x), \ldots, v_{k,a_k}(x)$ are the a_k successors of x in this updating.

3 MODEL MODIFICATIONS

Range restriction

We call $f_i^{a,b}$ the restriction of the function f_i to the range $[a, b]$ (with $0 \leq a \leq b \leq m_i$) such that:

$$f_i^{a,b}(x) = \begin{cases} a & f_i(x) < a \\ b & f_i(x) > b \\ f_i(x) & \text{otherwise: } a \leq f_i(x) \leq b \end{cases} .$$

Perturbation of an interaction

The removal of an interaction (i, j) (*i.e.* the removal of i from the regulators of j) further requires the specification of $v \in \mathcal{S}_i$ and leads to the modification of f_j, the logical rule associated to j.

Let $x^{i,v}$ be the state such that $x_i^{i,v} = v$ and $x_k^{i,v} = x_k \; \forall k \neq i$. The perturbation constructs the modified function $f_j^{i:v} = f_j(x^{i,v})$.
